# Perspective of synaptic protection after post-infarction treatment with statins

**DOI:** 10.1186/s12967-015-0472-6

**Published:** 2015-04-13

**Authors:** Johanna Andrea Gutiérrez-Vargas, Angel Cespedes-Rubio, Gloria Patricia Cardona-Gómez

**Affiliations:** Cellular and Molecular Neurobiology Area, Group of Neuroscience of Antioquia, School of Medicine, SIU, University of Antioquia UdeA, Calle 70 No. 52-21, Medellín, Colombia; Neurodegenerative Diseases Research Group, Department of Animal Health - Faculty of Veterinary Medicine - University of Tolima, Ibague, Colombia

**Keywords:** Cerebral ischemia, Statins, Synaptic proteins, Synaptic plasticity, Post-infarct protection

## Abstract

Stroke is the second most common cause of death in people over 45 years of age in Colombia and is the leading cause of permanent disability worldwide. Cerebral ischemia is a stroke characterized by decreased blood flow due to the occlusion of one or more cerebral arteries, which can cause memory problems and hemiplegia or paralysis, among other impairments. The literature contains hundreds of therapies (invasive and noninvasive) that exhibit a neuroprotective effect when evaluated in animal models. However, in clinical trials, most of these drugs do not reproduce the previously demonstrated neuroprotective property, and some even have adverse effects that had not previously been detected in animal experimentation.

Statins are drugs that inhibit 3-hydroxy-3-methylglutaryl coenzyme A (HMG-CoA) reductase, the rate-limiting enzyme in cholesterol synthesis. Several studies have shown that statin therapy in an animal model of focal cerebral ischemia reduces infarct volume, as well as markers of neurodegeneration, activates neuronal survival pathways, and improves performance on learning and memory tests. Given the implied therapeutic benefit and the limited understanding of the mechanism of action of statins in brain repair, it is necessary to address the biochemical and tissue effects of these drugs on synaptic proteins, such as NMDA receptors, synaptic adhesion proteins, and cytoskeletal proteins; these proteins are highly relevant therapeutic targets, which, in addition to giving a structural account of synaptic connectivity and function, are also indicators of cellular communication and the integrity of the blood–brain barrier, which are widely affected in the long term post-cerebral infarct but, interestingly, are protected by statins when administered during the acute phase.

## Introduction

Stroke is the third leading cause of death in industrialized countries [1] and the most common cause of disability in adults worldwide [2]. In Latin America, the main causes of the risk of death are cardiovascular accidents and stroke due to a sedentary lifestyle, diabetes, atherosclerosis, smoking, and hypertension. These diseases also increase the risk of developing neurodegenerative diseases, such as Alzheimer and vascular dementia [3].

Industrialized countries have turned their interest to the aforementioned health issues as increasing life expectancy has made them more prevalent in most of the world, becoming the silent epidemic of the current millennium. Developing countries are also increasing their life expectancy, and this disorder is beginning to become a public health problem with few current alternatives for healing.

## Cerebral ischemia

Cerebral ischemia is a type of stroke characterized by a transient or permanent reduction in blood flow due to embolic or thrombotic occlusion of one or more cerebral arteries [4,5]. Depending on the duration of the reduced blood flow and infarct location, cerebral ischemia can cause various clinical manifestations, including paralysis or hemiplegia, aphasia, and deficits in learning and memory processes, among other clinical manifestations [6].

If blood vessels that supply blood to all or most of the brain become clogged, the injury is called global ischemia, which usually happens during a heart attack or severe systemic hypotension [7]. However, if only the occlusion of vessels supplying blood to a certain area of the brain occurs, a focal ischemia is generated [8]. Approximately 80% of cases of focal ischemia result from occlusion of the middle cerebral artery (MCA) [9].

**Figure 1:**
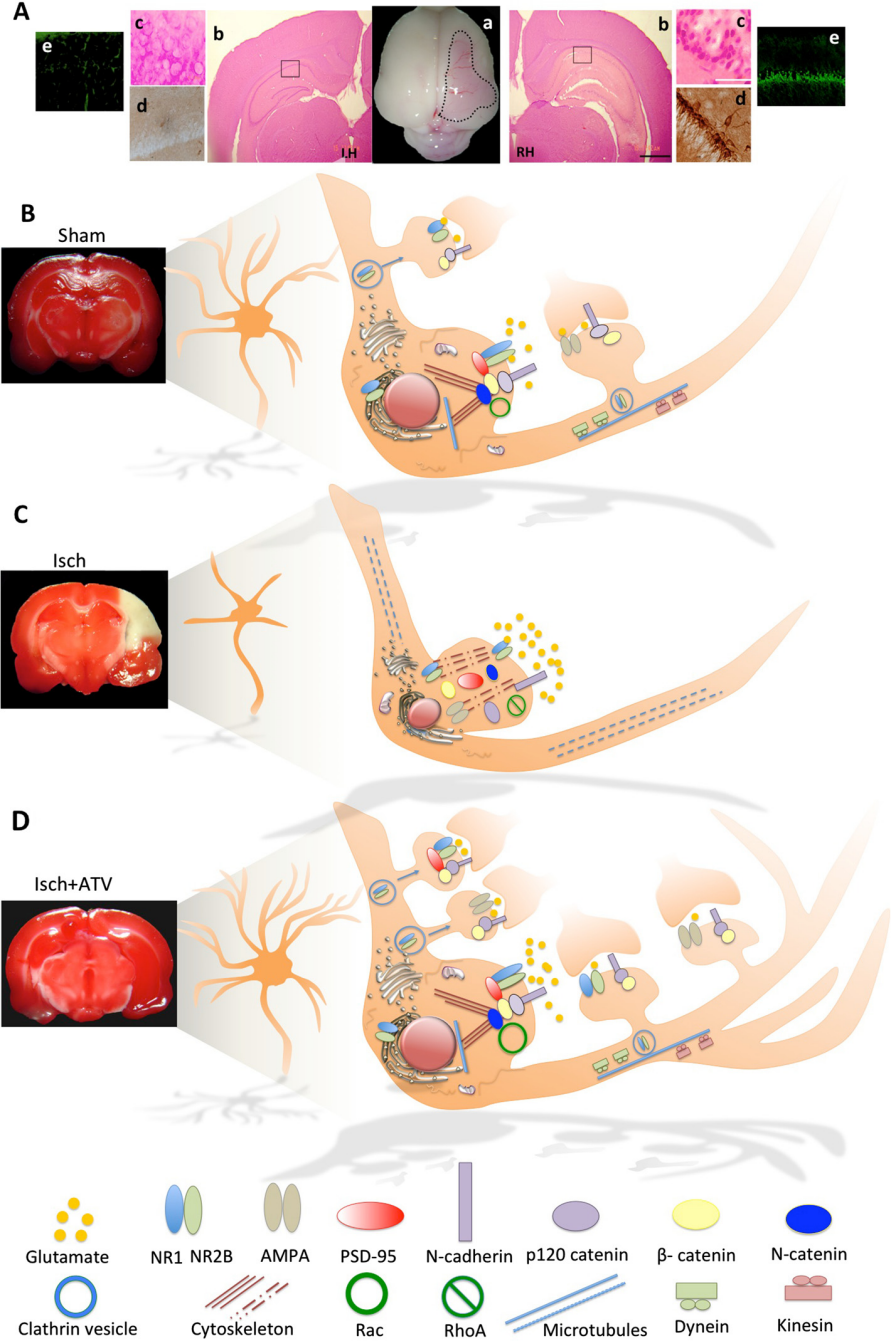
Neurodegeneration and protection in a focal cerebral ischemia model in rats. **A)** Histopathology of focal cerebral ischemia (tMCAO) model in rats. **a)** Panoramic representative image of a whole rat brain with tMCAO injury. **b)** Contralateral and ipsilateral images from cerebral ischemic rat tissue with Hematoxilin-eosin staining at the −3,80 mm bregma. LH = left hemisphere, RH = Right hemisphere, square = Selected area from CA1 area. Scale bar = 1,7 mm. **c), d), e)** Left side representative images from Sham rats and Right side representative images from ischemic rats. **c)** Hematoxilin-eosin staining 100x from CA1 square in **b)**. Scale bar = 50 μm. **d)** Hyperphosphorylated tau immunoreactivity (AT-8) staining 40X in CA1 area. Scale bar = 70 μm. **e)** Fluorojade staining 10x. Scale bar = 100 μm. **B)**, **C)** and **D)** Comparative hypothetical model of synaptic proteins alteration following cerebral ischemia and Atorvastatin treatment. **B)** Sham, **C)** Ischemia and D) ATV-treated ischemia. **B)** Synaptic complexes formed by proteins of synaptic adhesion (cadherins and catenins), glutamate receptors, and scaffold proteins, such as PSD-95 are associated in a normal condition (Sh = Sham). **C)** Glutamate receptors are uncoupled from PSD-95 and are accumulated in the cytoplasm due to retention of subunits in the endoplasmic reticulum (ER) because of the loss of transport toward synapses due to the alteration of microtubules and the cytoskeleton. Proteins that regulate the cytoskeleton, such as RhoA increased and synaptic complexes formed by the complex cadherins/catenins, glutamate receptors, and post-synaptic proteins, such as PSD-95 are lost. **D)** Model of protection by statins after cerebral ischemia. Synaptic complexes formed by proteins of synaptic adhesion (cadherins and catenins), glutamate receptors, and scaffold proteins, such as PSD-95 are restored in synapse, inducing neuronal connectivity. ISCH= Ischemia, ATV = Atorvastatin.

In focal ischemia, the region that suffers the most severe degree of hypoperfusion progresses rapidly towards irreversible damage due to a necrotic death, which represents the *ischemic core*. This area exhibits low cerebral blood flow (<10% of the baseline value) and the irreversible failure of energy metabolism [10]. The remaining hypoperfused tissue surrounding the ischemic core has an imbalance in the mechanisms of the autoregulation of blood flow and is known as the *penumbra zone* [11,12]. In this region, neurons show alterations in functionality, although they retain a minimum metabolic activity that preserves structural integrity for a longer period of time, following a pattern of apoptotic death (Figure 1A) [13,14]. The penumbra is potentially recoverable and, as such, represents a key target for therapeutic intervention in cerebral ischemia [15]; however, unless perfusion is improved or the cells become relatively resistant to the injury, the cells of the penumbra zone are at risk of dying within a few hours by necrosis [10,16].

## Excitotoxicity in cerebral ischemia

The interruption of blood flow into the brain due to ischemia results in the deprivation of oxygen and glucose, reducing the energy available for the operation of brain cells [8]. Neurons in particular become unable to maintain the ion gradients needed for cell function and homeostasis [17], which results in excessive neuronal depolarization, increased release of excitatory neurotransmitters, and reduced capacity of the reuptake of these neurotransmitters by astrocytes. The overloading of glutamate, the main excitatory neurotransmitter in the central nervous system (CNS) of mammals, leads to prolonged stimulation of AMPA ionotropic glutamate receptors (α-amino-3-hydroxy-5-methyl-4-isoxazolepropionic acid) and NMDA receptors (N-methyl-D-aspartic acid), dramatically increasing the influx of calcium (Ca^2+^), sodium (Na^+^), potassium (K^+^), and water into the neurons [18]. Excessive accumulation of ions and the simultaneous dysregulation of various signaling pathways activate catabolic processes mediated by proteases, lipases, and nucleases, which disrupt neuronal function and lead to cell death [8,17].

## Cerebral ischemia and alteration of synaptic proteins

### Ionotropic Receptors

**Figure 2:**
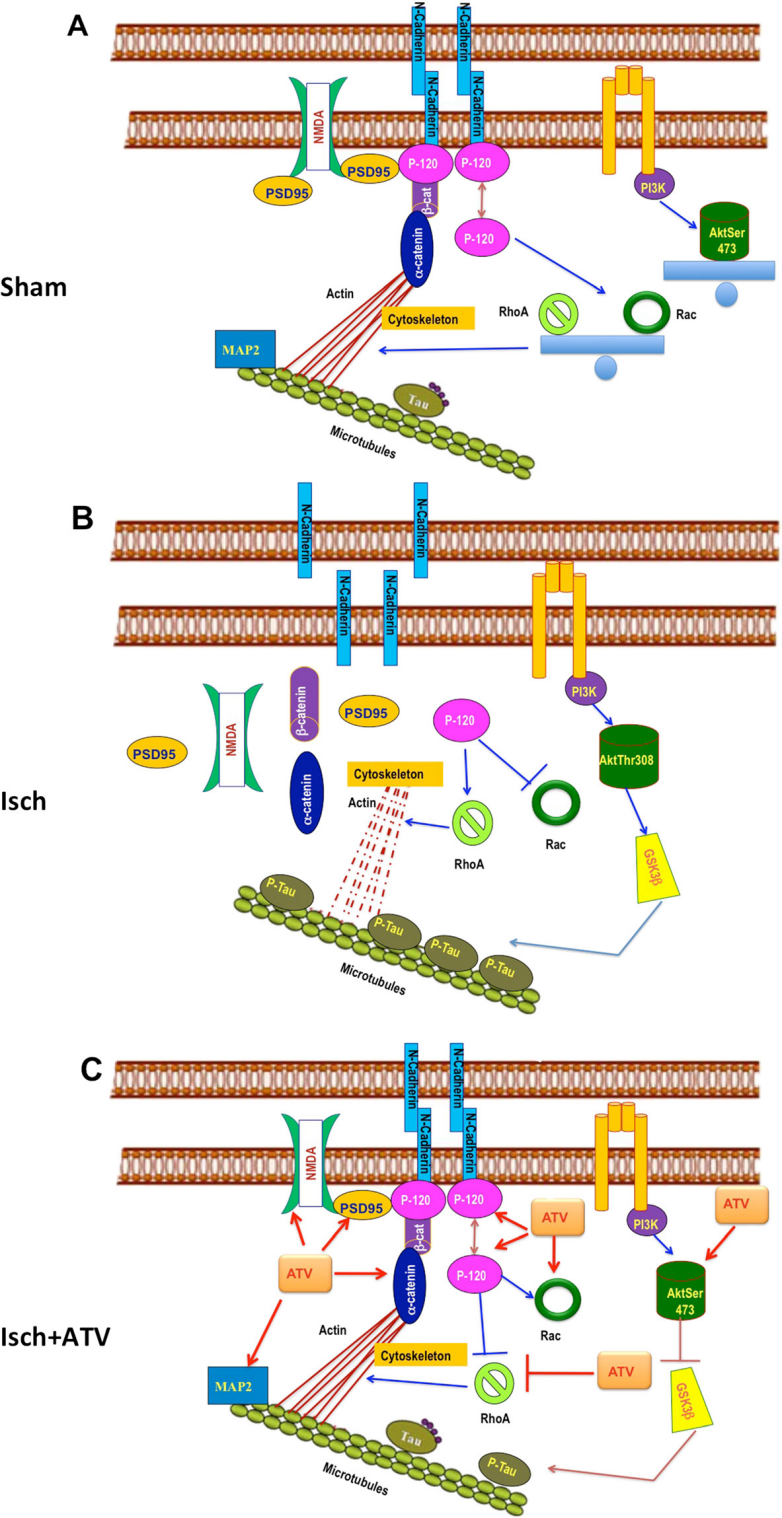
Schematic representations of cell signalling alteration following cerebral ischemia and Atorvastatin treatment. **A)** Sham, **B)** Ischemia and **C)** ATV-treated ischemia. **A)** NMDA receptors, synaptic adhesion proteins and PSD-95 are associated in the synapse. The stability of the actin cytoskeleton is regulated by the RhoGTPasas (Rac, RhoA) balance and microtubules by protein such as MAP2. **B)** After cerebral ischemia cell adhesion is lost by alteration in the cadherin-catenin complex; NMDA receptors and PSD-95 protein are distributed to the cytoplasm. The increased activity of RhoA, destabilizes actin cytoskeleton and GSK3b activation leads to tau hyperphosphorylation disassembling microtubules. **C)** Treatment with atorvastatin recovers the adhesion protein complex as well as the location NMDA receptor synapses associated to PSD-95. Additionally atorvastatin leads to AKT activation promoting cell survival. Recovery of the actin cytoskeleton is mediated by the increased activity of Rac and RhoA reduction, and finally atorvastatin stabilize microtubules.

After the establishment of neuronal connections during development, the synapse remains highly dynamic and undergoes changes in morphology and efficiency, which are dependent on activity. The presynaptic terminal includes the machinery for neurotransmitter release, while post-synaptic sites include proteins such as neurotransmitter receptors and signaling proteins that promote the response to the released neurotransmitter, which allows the transmission of information (Figures 1B, 2A) [19,20].

Among neurotransmitter receptors, NMDA receptors have been extensively studied because they represent the largest subclass of glutamate receptors in mammalian excitatory synapses. They consist of four subunits, two of which are NR1 combined with NR2 (NR2A-D) or NR3 (NR3A-B) subunits [21]. These receptors play a central role in synaptic transmission, neuronal plasticity, regulation of neuronal development, and connectivity, as well as in memory and learning processes, primarily due to their permeability to Ca^2+^ [22-24].

Located at the synapses, the NMDA receptors are not static proteins; in fact, the number and composition of NMDA receptors can be modified, which provides a dynamic mechanism for the regulation of synaptic efficacy and remodeling [25]. Different regions of the receptor, as well as scaffold proteins, such as PSD-95, and other proteins of the postsynaptic density, all participate in the mechanisms of internalization of NMDA. NMDA receptors that bind to PSD-95 are stable in the membrane and are less likely to be internalized (Figures 1B, 2A), while receptors that are not bound to proteins of the postsynaptic density can be readily internalized. This mechanism can “remove” from the membrane surface NMDA receptors that are not permanent at the synapse [26].

The overactivation of NMDA receptors in cerebral ischemia induces excitotoxic cell death. Excessive activation of NMDA induces a rapid and specific transcriptional inhibition of the NR1 subunit promoter in a process that is strictly dependent on Ca^2+^ influx through the receptor. NR1 transcriptional inhibition results in a progressive reduction of its mRNA, which leads to a progressive reduction of the protein [27]. Overstimulation of NMDA receptors can lead to a progressive decline in receptor activity because its functionality depends strictly on the expression of hetero-oligomers NR1/NR2 on the cell surface [28].

Studies have shown that NR2A and NR2B subunits play different roles in excitotoxic cell death [29]. While inhibition of NR2B induces cell death and prevents motor and cognitive recovery [30], inhibition of the NR2A subunit leads to cell survival in a model of cerebral ischemia [29]. In models of excitotoxicity, it has been seen that excessive activation of the NMDA receptors leads to increased intracellular calcium, which results in the activation of calpain, a protease that mediates cellular toxicity [31,32]. This protease efficiently cuts the C-terminal region of the NR2A and NR2B subunit, producing truncated subunits that lack certain sequences near serine-1303, which are critical to the formation of signaling complexes in the postsynaptic membrane [33]. The end result is the uncoupling of NMDA receptors from scaffolding proteins, such as PSD-95 (Figures 1C, 2B), affecting synaptic function [32].

The results obtained in our research group show that subunits of NMDA receptors are expressed in the cytoplasm following ischemia [30], which may be related to a deficiency in the assembly of the different subunits making up the receiver and/or problems in the transport from the receptor to the synapses (Figure 1C). In the *knockout* mouse for NR1, both reduction in the dendritic distribution and accumulation in the cytoplasm of the NR2B subunit [34] are observed, perhaps because of the loss of the assembly of functional NMDA receptors to the synaptic membrane or because NMDA receptors with NR2B subunits located in the synaptic density are internalized by endocytosis to perform autoregulation and avoid the continuous influx of calcium. It is known that clathrin-mediated endocytosis is a mechanism that regulates the synaptic expression of NMDA receptors containing NR2B during synaptic maturation in processes such as long-term potentiation (LTP) or long-term depression (LTD) as well as in response to a ligand [35].

## Cytoskeletal proteins and cell adhesion

Transport from the receptor to and from synaptic sites may be lost due to alteration of the microtubules and damage to the actin cytoskeleton [36] (Figure 1C), microtubule-associated proteins (MAP2) involved in the regulation of vesicle transport during development or regeneration of neuronal processes [37]. Our work and other previous studies have found that these proteins are redistributed in the soma after the acute post-ischemia period [38,39], which is considered an indicator of excitotoxic damage and loss of microtubule stability [38,39] (Figure 1C). The actin cytoskeleton is primarily mediated by the Rho GTPases, proteins that regulate its maintenance, remodeling, and stability [40]. The RhoA and Rac1 proteins are among the most studied of the Rho GTPases. In general terms, RhoA regulates actin assembly associated with processes of cellular shrinkage due to stress [41,42], while Rac regulates actin polymerization, which is related to axonal growth and stability and the increase of dendritic processes [41,43]. Our research shows that RhoA activity is increased in excitotoxic death processes [44] (Figures 1C, 2B), and inhibiting this activity reduces markers of neurodegeneration as well as improves learning and memory processes in cerebral ischemia [45]. Complementarily, in the acute post-ischemia period (24 hours), Rac1 activity decreases and this protein is redistributed in the soma and in aberrant neuronal processes, which forms part of the process of chronic neurodegeneration [38].

Disassembly of the cytoskeleton in cerebral ischemia mediated by RhoA activation and inactivation of Rac is associated with the disruption of synaptic adhesion mediated by the complex of cadherin/catenins and [40], proteins that are present in both presynaptic and postsynaptic sites. Cadherins promote cell adhesion, and their association with catenins allows the control of different intracellular signaling pathways [19,46,47]. Structurally, cadherins have an extracellular domain comprising most of the polypeptide chain, generally composed of five repetitions, four of which are capable of binding extracellular calcium. This extracellular domain mediates calcium-dependent homophilic interactions with cadherins of adjacent neurons. The cytoplasmic region includes the binding sites to a variety of proteins, including catenins [48]. Catenins are cytosolic proteins that are basically subdivided into three groups: β-catenin, α N-catenin, and p120 catenin (p120 ctn). The distal region of the cadherin’s cytoplasmic domain includes the binding site for β-catenin, while the nearest region to the membrane contains the binding site for family members of p120 ctn. The protein α N-catenin binds to the complex by binding with β-catenin [19].

In our recent studies using a model of focal ischemia in rats, the levels of N-cadherin, p120 catenin, and ∝N catenin in the cerebral cortex and hippocampus were significantly reduced, as was their association with PSD-95 and glutamate receptors (NMDA and AMPA) (Figures 1C, 2B), which represents the loss of synaptic adhesion and of complexes associated with synaptic plasticity [44]. Additionally, there was an interdependence between Rac activity and the ∝N catenin distribution in dendrites, which was lost in the event of glutamate excitotoxicity in primary neuronal cultures [49].

Glycogen synthase kinase-3β (GSK-3β) is an essential protein downstream of Akt that is involved in the pathological process of brain damage. The PI3K/Akt signal transduction pathway may affect the activity of GSK-3β. Active Akt inhibits GSK-3β activity via phosphorylation [50]. Sustained activation of GSK-3β is pro-apoptotic in cerebral ischemia, because this leads to hyperphosphorylation of tau, microtubule destabilization (Figure 2B) and cognitive impairment.

The foregoing are some of the cellular and biochemical events underlying the impairment and neuronal death after cerebral ischemia; however, it is important to understand the precise mechanisms of the proteins involved in the synapse, and their implication in morphological and functional recovery. Such knowledge is invaluable in the development of effective neuroprotective strategies to achieve an improved clinical outcome, which results not only in survival but also in a higher quality of life for patients who suffer acute cerebrovascular events, and to reduce or prevent long-term physical and mental consequences.

### Statins as treatment after a cerebral ischemia

The pharmacological approach for inducing neuroprotection after an injury due to cerebral ischemia aims at blocking signaling pathways that trigger cell death, to induce synaptic connectivity, and functional recovery. The main goal is to limit the devastating consequences of reduced blood flow in the penumbra zone induced by reperfusion [14,51].

Statins are the drugs of choice for the treatment of hypercholesterolemia that lower low density lipoproteins (LDL) and triglycerides [52]. The mechanism of action of these drugs is via competitive and selective inhibition of 3-hydroxy-3-methylglutaryl coenzyme A (HMG-CoA) reductase, the rate-limiting enzyme in cholesterol synthesis because it is responsible for the conversion of 3-hydroxy-3-methylglutaryl CoA to mevalonate, which is a precursor of cholesterol [52,53]. Studies such as the *Heart Protection Study*, in which statin therapy significantly reduces the coronary mortality rate and incidence of stroke, confirm the need for strict control of the lipid profile to reduce the overall risk and the mortality rate caused by cardiovascular diseases and stroke [54]. Also, a recently completed clinical trial, NCT02225834 in phase IV, used Atorvastatin as acute stroke treatment to evaluate separate effects on immunoinflamatory markers and atherothorombosis; or a recently terminated clinical trial, NCT01364220 in phase III, used Rosuvastatin on early window for preventing recurrence of ischemic stroke.

However, in addition to their effect on the lipid profile, statins are also credited with effects that are independent of the reduction of cholesterol synthesis, called *pleiotropic effects*. By inhibiting the conversion of hydroxy-methylglutaryl Coenzyme A (HMG-CoA) to L-mevalonate, statins block the synthesis of important isoprenoids, such as farnesyl pyrophosphate (FPP) and geranylgeranyl pyrophosphate (GGPP), which are important intermediates for the post-translational modification of Rho GTPases (RhoA, Rac, and Cdc42) [53]. Among the *pleiotropic effects* reported in cerebral ischemia are the improvement of endothelial function, the stability of the atherosclerotic plaque, the attenuation of oxidative stress and inflammation, and the inhibition of the thrombogenic response [53]. Previous studies conducted on cerebral ischemia showed that statins improve neurological function and increase cerebral blood flow [55-57], mitigating the effects of inflammation and post-traumatic hypoperfusion [53], in addition to protecting blood vessels and promoting the processes of angiogenesis, neurogenesis, and synaptogenesis after cerebral ischemia [58].

Using an embolic model, simvastatin-treated rats showed a significant infarct volume reduction and neurological improvement compared to vehicle-treated group. Analyzing their homogenated brains by two-dimensional fluorescence Difference in Gel Electrophoresis (DIGE) technology, observed that the protective effect of simvastatin can be attributable to oxidative stress response attenuation and blood–brain barrier protection after cerebral ischemia [59]. There are some additional evidences in humans, where the treatment with statins is not only associated to a reduced incidence of stroke but with reduced stroke severity, as well [60]. In a small pilot trial with simvastatin in acute ischemic stroke (AIS), Montaner et al. showed more cases of complete functional recovery after 3 months of treatment with simvastatin (40 mg/day) in comparison with the non-treated group (p < 0.05). The significant improvement was observed on the third day of treatment [61].

A study by Kawashima et al. showed that cerivastatin reduce the stroke-associated infiltration of inflammatory cells, superoxide production in the cerebral parenchyma, and cerebral damage caused by stroke, as well as delay the appearance of stroke-associated symptoms and death in stroke-prone, spontaneously hypertensive rats [62]. Recently, we found that atorvastatin treatment increases the expression of brain-derived neurotrophic factor (BDNF) in an NR2B-dependent manner [30]; this trophic factor is involved in survival [63] and is correlated with motor recovery and with spatial learning and memory (29). We also confirmed that statins activate the PI3K/Akt pathway (Figure 2C) and Ras/Erk, which transduce survival signals and in turn promote synaptic plasticity [44].

### Effect of statins on synaptic proteins

In an experimental model of focal cerebral ischemia, we describe that in addition to neurological recovery and the reduction of infarct volume and of neurodegeneration markers, statin therapy during the acute post-ischemia phase achieves the restoration of proteins involved in neuronal connectivity, such as NMDA receptors [30], synaptic adhesion proteins (cadherins and catenins) and cytoskeletal proteins (RhoA and Rac) [44]. Atorvastatin restores the levels of N-cadherin, p120 catenin and ∝N catenin protein in the cerebral cortex and in the hippocampus along with the association of this complex of cadherins/catenins with the PSD-95 protein (Figure 1D) [44]. These findings support the restoration of synaptic complexes in cerebral ischemia following statin therapy. Additionally, we have found that atorvastatin treatment prevents the cytoplasmic distribution of subunits of glutamate receptors (NR1, NR2B NMDA) and recovers their association with PSD-95 and with proteins of synaptic adhesion (Figure 1D) [30]. The latter is related to the recovery of microtubule stability due to statin therapy, as MAP2 immunoreactivity is restored both in the somatosensory cortex and in the hippocampus [30,44], enabling the transport of the receptors and their stability at the synapses (Figure 1 D). The recovery of synaptic molecular complexes that involve NMDA receptors and synaptic adhesion proteins is correlated with a recovery of the motor and spatial learning and memory deficits caused by ischemia [30].

Complementarily, we describe that atorvastatin postischemia treatment tended to reduce RhoA activity which leads to recovery the actin cytoskeleton (Figure 2C) [44]. By down-regulating Rho/Rho kinase signaling pathways, statins increase the stability of eNOS mRNA and induce activation of eNOS through phosphatidylinositol 3-kinase/Akt/eNOS pathway restoring endothelial function [64]. The beneficial effects of Akt activation are not limited to eNOS phoshorylation but extend to the promotion of survival neuronal (Figure 2C).

### Perspective

It is becoming necessary to advance both theoretically and experimentally in the study of the factors involved in cerebral ischemia and in the rigorous evaluation of the drugs to be used and, with these results, to be able to impact more precisely a population at increased risk of suffering stroke. The purpose is to prevent, block, or meet the needs of the affected community not only in the short-term but also in the medium- and long-term post-cerebral infarct period. This development must be accompanied by training and improving the expertise necessary for diagnosis and for making decisions in clinical treatment inside and outside the therapeutic window as well as by prevention programs designed to reduce neurodegenerative disorders in general and to improve quality of life and access to health services.

According to the above, our work has specifically contributed to elucidating the mechanisms of pathogenesis and therapy in cerebral ischemia as well as to the understanding of the mechanisms and the cellular and molecular targets of protection by statins. Our previous results and the issues raised in this review allow us to recommend from preclinical study in rats that atorvastatin, a drug already on the market for human consumption as a treatment for hypercholesterolemia, could be used as a protective treatment during the first 6 hours post-ischemic infarct. Its administration for at least three days and ideally longer, with attention to the patient’s adherence to the drug and its quality, could potentially reduce or prevent the medium- and long-term severity of neurologic dysfunction and of emotional and cognitive compromise in patients.

## Conclusion

In summary, statin therapy after a cerebral infarct blocks the spread of damage and helps to restore brain morphology and functionality, at least based on the induction of trophic factors such as BDNF and of cell adhesion proteins that generate a platform for synaptic proteins, such as PSD-95, and NMDA receptors to settle and generate synaptic connectivity, neurotransmission, and cognitive and motor function recovery.
